# Multidimensional impulsivity as a mediator of early life stress and alcohol dependence

**DOI:** 10.1038/s41598-018-22474-8

**Published:** 2018-03-07

**Authors:** Shin Tae Kim, Syung Shick Hwang, Hae Won Kim, Eun Hee Hwang, Jaeil Cho, Jee In Kang, Se Joo Kim

**Affiliations:** 1 Institute of Behavioral Science in Medicine, Yonsei University College of Medicine, Seoul, South Korea; 2 Graduate School, Yonsei University College of Medicine, Seoul, South Korea; 3 Yonsei Soul Mental Health Clinic, Seoul, South Korea; 4 Department of Psychiatry, Yonsei University College of Medicine, Seoul, South Korea

## Abstract

Early life stress (ELS) leads to increased susceptibility to serious psychiatric problems such as alcohol dependence, but the mechanisms through which ELS affects alcohol dependence are unclear. We investigated the mediating role of multi-dimensional impulsivity in the associations between ELS and alcohol dependence. 330 male patients with alcohol dependence (mean age = 48.39) completed self-rating scales of ELS and several self-report measures of impulsivity as well as balloon analogue risk task (BART). After classifying different dimensions of impulsivity using factor analysis, structural equation modeling was conducted to test the mediation effects of impulsivity between ELS and alcohol dependence severity and social onset of hazardous drinking. Among the participants, 64.8%, 42.1% and 47.9% reported at least one episode of childhood maltreatment, sexual abuse and parental conflict, respectively. Response impulsivity-sensation seeking, reflection impulsivity and aggression partially mediated the association between ELS and severity of alcohol dependence (CFI = 0.902 and RMSEA = 0.079). Reflection impulsivity dimension partially mediated the association between ELS and social onset of hazardous drinking (CFI = 0.939, RMSEA = 0.091). These finding imply that stabilizing vulnerabilities such as reflection impulsivity via intervention programs that target young individuals with ELS may be helpful in delaying the onset of hazardous drinking and prevent alcohol dependence.

## Introduction

Alcohol use disorder (AUD) is an important mental, physical and public problem with high social and economic burden^1,2^. Harmful alcohol use was ranked as the leading risk factor for disease, injury and disability throughout the world and it was reported to account for 5.9% of all deaths worldwide^3,4^. Furthermore, alcohol-related family disruption, violence and criminal behaviors lead to serious public health problems^5,6^. Given these high burdens associated with excessive alcohol consumption, it is very important to understand the risk factors and their nature associated with alcohol use disorder and to develop intervention strategies for prevention of problematic alcohol use.

Early life stress (ELS) is an important risk factor that confers increased vulnerability for problematic alcohol use. Substantial evidence supports the risky relationship between ELS and alcohol consumption. A large epidemiology study of 17,337 HMO members showed that there was a graded relationship between the number of adverse childhood experiences and the risk for alcohol dependence; people who experienced four or more categories of ELS were at a 7.2-fold increased risk for alcohol use disorder, compared to people without any experience of ELS after controlling for demographic factors^7^. In addition, alcoholics with a history of childhood trauma were more likely to attempt suicide^8^. Furthermore, adverse childhood experience was found to be associated with earlier initiation of alcohol use during adolescence^9^. A study of 3,592 US adults showed those with a history of four or more types of ELS had an increased risk of 3.6 times the odds for initiating drinking at the age of 14 or younger and 1.8 times at the ages from 15 to 17, compared to those without any experience of ELS, when adjusted for multiple variables including family feelings about alcohol and number of friends drinking first year of high school^10^. These findings suggest that ELS is a significant risk factor for development and prognosis of problematic alcohol drinking. While growing evidence supports the relationship between ELS and alcohol use, the mechanisms for how ELS affects problematic alcohol use later in adulthood are not yet well understood. From a neurodevelopmental perspective, ELS may lead to negative cognitive and affective sequalae such as impaired executive function and emotional regulation through neurodevelopmental alteration, contributing to vulnerability to risky behaviors and psychiatric disorders^11^. Brain imaging studies support that childhood trauma leads to long lasting neural changes in brain regions involving emotion regulation and self-control later in life^12,13^. In particular, impulsivity, characterized by the lack of self-control and the inability to wait for delayed gratification^14^, may play a key role in the link between ELS and alcohol dependence, since impulsivity is a major risk factor for addiction^15^ and its development is influenced by environmental factors such as childhood adverse experiences^16,17^. A recent study with a community sample aged 18–25 reported that negative urgency, a subdimension of impulsivity associated with failure of self-control under negative emotion, may play a mediating role between childhood emotional abuse and frequency of alcohol use, binge drinking and alcohol use disorder^18^.

Impulsivity is a complex and multi-dimensional trait^19,20^. It includes facets such as reflection impulsivity (the tendency to act quickly without sufficiently evaluating pertinent information), response disinhibition (the predisposition to react urgently with inability to inhibit undesirable thoughts and actions), sensation and novelty seeking (the tendency to chase novel or thrilling activities) and risk taking (the predisposition to choose risky options with immediate reward)^20^. A meta-analytic review on multi-dimensional impulsivity traits and alcohol use found that the impulsivity dimension of acting urgently in response to emotional states had the strongest association with problematic alcohol use^21^. Because several discrete impulsivity traits may influence the course of alcohol use disorder through different pathways, a comprehensive model which includes various constructs of impulsivity is necessary to better understand the role of impulsivity through which ELS contributes to adulthood alcohol use.

The present study aimed to investigate the relationship between ELS, multi-dimensional impulsivity and alcohol problems in Korean male patients with alcohol dependence. Here, we focused on the mediating role of multi-dimensional impulsivity in the associations between ELS and alcohol dependence severity and between ELS and onset of hazardous drinking, using structural equation modeling.

## Results

The demographic and clinical characteristics of the patients are presented in Table 1. Among patients with alcohol dependence, 64.8%, 42.1% and 47.9% reported at least one episode of childhood maltreatment, sexual abuse and parental conflict, respectively and 21.2% reported experience of all three types.

**Table 1:** Demographic and Clinical Characteristics of the Patients.

Variable		N(%) or Mean ± SD
Participants		n = 330
Age		48.39 ± 7.91
Social onset of hazardous drinking		30.97 ± 9.93
Duration of illness, years		17.42 ± 10.37
Alcohol dependence severity	AUDIT	26.63 ± 7.67
	OCDS	19.16 ± 7.28
	ADS	21.23 ± 10.41
Early life stress	Sexual abuse scale	4.33 ± 8.87
	mPCCTS	68.06 ± 83.09
	mCTS	4.50 ± 6.41

mPCCTS: modified Parent-Child Conflict Tactics Scale; mCTS: modified Conflict Tactics Scale; AUDIT: Alcohol Use Disorders Identification Test; OCDS: Obsessive Compulsive Drinking Scale; ADS: Alcohol Dependence Scale.

From the factor analysis, four impulsivity dimensions were extracted. Factor 1 included BIS: Non-planning, BIS: Attentional, BIS: Motor, UPPS: Perseverance and UPPS: Pre-planning, which was named “Reflection Impulsivity”^22^. Factor 2 included UPPS: Positive Urgency, UPPS: Negative Urgency and UPPS: Sensation Seeking, which was named “Response Impulsivity-Sensation Seeking”. Factor 3 included only the BART, which was named “Risk Taking”. Factor 4 included BPAQ: Physical Aggression, BPAQ: Verbal Aggression, BPAQ: Anger and BPAQ: Hostility, which was named “Aggression”. The Cronbach’s α and factor loading values are presented in Table 2. In addition, factor score for each variable and its correlations with observed variables regarding ELS and alcohol problems are presented in Supplementary Table 1.

**Table 2:** Factor loadings of multi-dimensional impulsivity.

Factors	Mean ± SD	Factor Loading
**Factor 1: Reflection Impulsivity (α = 0.875)**		
UPPS Pre-Planning	21.98 ± 5.05	0.768
UPPS Perseverance	20.79 ± 4.71	0.843
BIS Attentional	14.49 ± 3.29	0.825
BIS Motor	15.75 ± 4.43	0.756
BIS Non-Planning	20.62 ± 4.68	0.859
**Factor 2: Response Impulsivity-Sensation Seeking (α = 0.752)**		
UPPS Positive Urgency	36.88 ± 6.53	0.894
UPPS Negative Urgency	31.20 ± 5.72	0.709
UPPS Sensation Seeking	29.70 ± 6.85	0.718
**Factor 3: Risk Taking**		
BART (Adjusted mean numbers of pumps)	28.98 ± 17.00	0.984
**Factor 4: Aggression (α = 0.825)**		
BPAQ Physical Aggression	21.82 ± 6.74	0.787
BPAQ Anger	18.10 ± 5.25	0.834
BPAQ Hostility	18.61 ± 6.31	0.745
BPAQ Verbal Aggression	13.05 ± 3.88	0.796

UPPS: UPPS Impulsive Behavior Scale; BIS: Barratt Impulsiveness Scale; BART: Balloon Analogue Risk Task; BPAQ: Buss-Perry Aggression Questionnaire.

**Figure 1:**
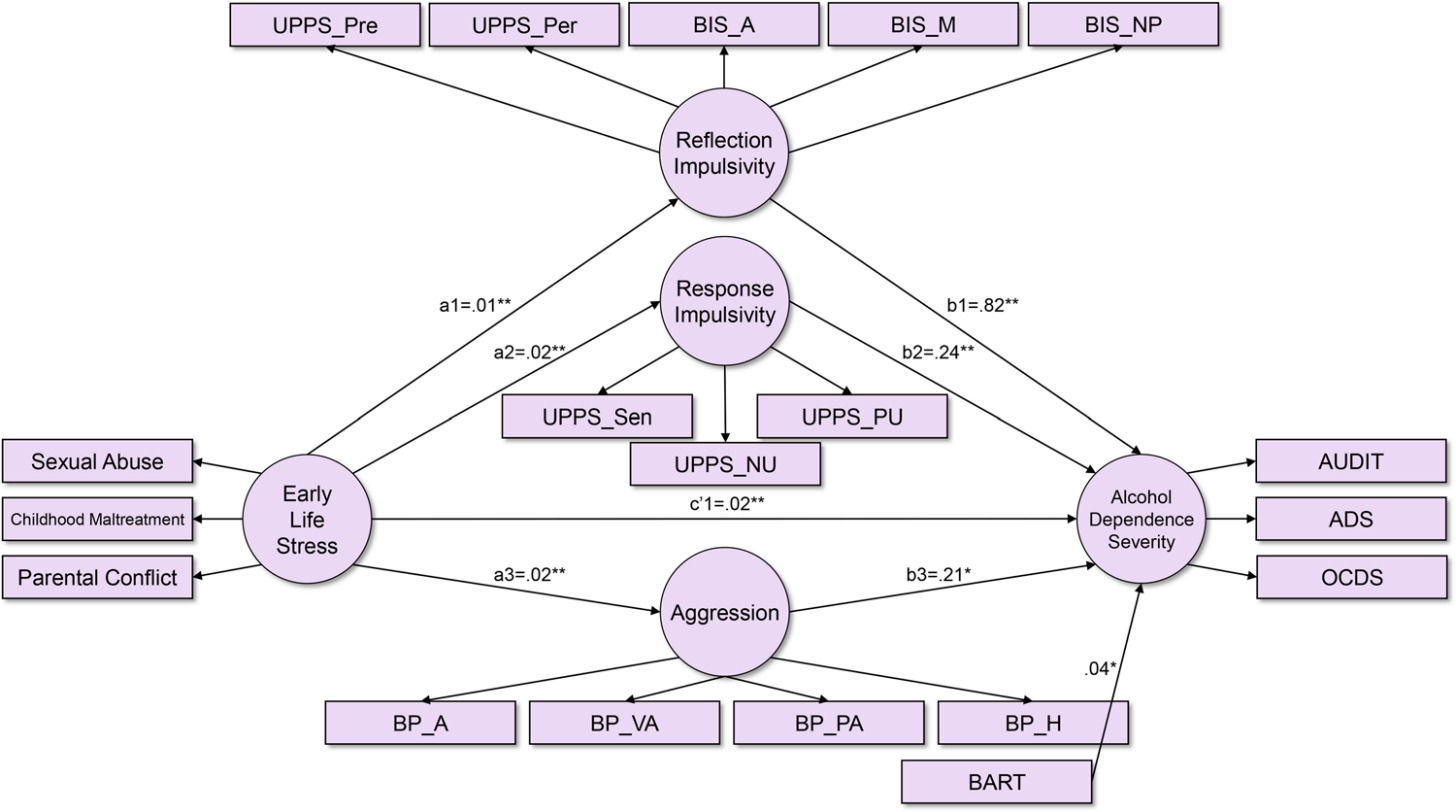
Model of early life stress, multi-dimensional impulsivity and alcohol dependence severity (Mediation Model A). Coefficients are unstandardized estimates. *p < 0.05, **p < 0.01. Parental Conflict: modified Conflict Tactics Scale; Childhood Maltreatment: Modified Parent-Child Conflict Tactics Scale; Sexual Abuse: “Sexual abuse” section of the Childhood Maltreatment Scale; AUDIT: Alcohol Use Disorders Identification Test; OCDS: Obsessive Compulsive Drinking Scale; ADS: Alcohol Dependence Scale; UPPS: UPPS Impulsive Behavior Scale; BIS: Barratt Impulsiveness Scale; Pre: Lack of Pre-planning; Per: Lack of Perseverance; A: Attentional; M: Motor; NP: Non-planning; NU: Negative Urgency; PU: Positive Urgency; Sen: Sensation Seeking; BART: Balloon Analogue Risk Task; PA: Physical Aggression; VA: Verbal Aggression; A: Anger; H: Hostility.

The direct effect model of “ELS” and “Alcohol Dependence Severity” (Direct Effect Model A) provided good model fit (CFI = 0.989 and RMSEA = 0.056). In the direct effect model A, there was a significant relationship between “ELS” and “Alcohol Dependence Severity” (c1 = 0.035, p < 0.001). This model explained 14.8% of variance in “Alcohol Dependence Severity.” The mediation model A, which tested the mediating effects of four impulsivity dimensions in the relationship between “ELS” and “Alcohol Dependence Severity”, provided reasonable model fit (CFI = 0.902 and RMSEA = 0.079). However, since inspection of the model revealed that the path from “ELS” to “Risk Taking” was not significant, it was trimmed. The final mediation model provided reasonable model fit for the data (Fig. 1, CFI = 0.902 and RMSEA = 0.079). There was a significant relationship between “ELS” and “Alcohol Dependence Severity,” whose effect estimate (c’1 = 0.017, p < 0.01) was smaller than that in the direct effect model (c1 = 0.035, p < 0.001). In addition, all the paths connecting “ELS,” the mediator variables and “Alcohol Dependence Severity” were significant and bootstrapping revealed that the indirect effect of “ELS” on “Alcohol Dependence Severity” was significant (ab = 0.019, p < 0.001). The mediation effects of “Reflection Impulsivity,” “Response Impulsivity-Sensation Seeking,” and “Aggression” were also significant on their own and are presented in Fig. 1 and Table 3. This model explained 49.9% of variance in “Alcohol Dependence Severity.” The standardized regression weights for all the parameters in the mediation model A are presented in the Supplementary Table 2a.

**Table 3:** Mediation effects of multi-dimensional impulsivity in the relationships between early life stress and alcohol problems of symptom severity and social onset of hazardous drinking.

Mediating variable (M)	Effect of ELS on M (a)	Effect of M on Outcome variable (b)	Direct effect of ELS on Outcome variable (c’)	Indirect effect (a × b)
**Outcome: Alcohol Dependence Severity**				
Response Impulsivity-Sensation Seeking	0.022***	0.237**	0.017**	0.005**
Reflection Impulsivity	0.011***	0.823***		0.009**
Aggression	0.019***	0.214*		0.004**
**Outcome: Social Onset**				
Reflection Impulsivity	0.011***	−0.417*	−0.026**	−0.005*

ELS: Early life stress; *p < 0.05, **p < 0.01, ***p < 0.001.

The numbers presented are unstandardized coefficients.

**Figure 2:**
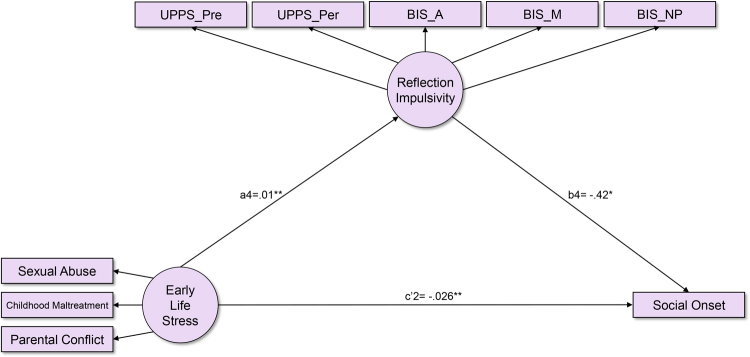
Model of early life stress, multi-dimensional impulsivity and social onset (Mediation Model B). Coefficients are unstandardized estimates. *p < 0.05, **p < 0.01. Parental Conflict: modified Conflict Tactics Scale; Childhood Maltreatment: Modified Parent-Child Conflict Tactics Scale; Sexual Abuse: “Sexual abuse” section of the Childhood Maltreatment Scale; Social Onset: age at the time when drinking started to cause social problems; UPPS: UPPS Impulsive Behavior Scale; BIS: Barratt Impulsiveness Scale; Pre: Lack of Pre-planning; Per: Lack of Perseverance; A: Attentional; M: Motor; NP: Non-planning.

On the other hand, for the “Social Onset”, the direct effect model of “ELS” (Direct Effect Model B) provided good model fit for the data (CFI = 0.985 and RMSEA = 0.067). In this direct model, there was a significant relationship between “ELS” and “Social Onset” (c2 = −0.03, p < 0.001) and the model explained 4.7% of variance in “Social Onset.” The mediation model B, the mediating effects of four impulsivity dimensions in the relationship between “ELS” and “Social Onset”, did not provide acceptable goodness of fit (CFI = 0.874 and RMSEA = 0.089). The paths from “Response Impulsivity-Sensation Seeking,” “Aggression,” and “Risk Taking” to “Social Onset” were not significant, so those latent variables were erased. The final mediation model B provided reasonable model fit for the data (Fig. 2, CFI = 0.939 and RMSEA = 0.091). There was a significant relationship between “ELS” and “Social Onset,” whose effect estimate (c’2 = −0.026, p < 0.01) was smaller than that in the direct effect model (c2 = −0.03, p < 0.001). In addition, the paths connecting “ELS,” “Reflection Impulsivity,” and “Social Onset” were significant and bootstrapping revealed that the indirect effect of “ELS” mediated through “Reflection Impulsivity” was significant (ab = −0.005, p < 0.05). The mediation effect of reflection impulsivity is presented in Fig. 2 and Table 3. This model shows that the relationship between “ELS” and “Social Onset” was partially mediated by “Reflection Impulsivity” This model explained 6.8% of variance in “Social Onset.” The standardized regression weights for all the parameters in the mediation model B are presented in Supplementary Table 2b.

## Discussion

The present study investigated the mediating role of multi-dimensional impulsivity in the associations between ELS and alcohol dependence severity and between ELS and social onset of problematic alcohol use in patients diagnosed with alcohol dependence using structural equation modeling. Response impulsivity-sensation seeking, reflection impulsivity and aggression partially mediated the association between ELS and alcohol dependence severity, while only reflection impulsivity partially mediated the association between ELS and social onset of hazardous drinking. Our results indicate that specific impulsivity dimensions may play a crucial role in the associations between trauma exposure in childhood and clinical course of alcohol dependence in adulthood.

Among impulsivity dimensions, response impulsivity-sensation seeking, reflection impulsivity and aggression, but not risk taking (BART), partially mediated the relationship between ELS and alcohol dependence severity (ab = 0.019, p < 0.001). Several reports support the role of impulsivity as a mechanism linking ELS and alcohol use later in life, although the applied concept and assessment methods of impulsivity (general vs. multifaceted) and sample characteristics (community sample vs. drinkers vs. clinical patients) are diverse among studies^18,23,24^. A report on a nationally representative sample revealed that negative urgency, positive urgency and sensation seeking dimensions of the UPPS, which corresponds to response impulsivity-sensation seeking in our study, indirectly connected childhood trauma to alcohol and cannabis use^24^. In addition, a recent study with a community sample of young adults showed that negative urgency subdimension of UPPS significantly mediates the relationship between childhood emotional abuse and alcohol use outcomes^18^. Response impulsivity trait which includes negative urgency may play an important role in urgent engagement in alcohol drinking in response to emotion or stressors for people with ELS. It can be explained by a neuroadaptive perspective on brain stress systems in which ELS exacerbates stress reactivity and failure of the inhibitory processes over limbic hyperresponsivity, consequently leading to substance use under stressful situations^12,13,25^.

On the other hand, risk taking impulsivity as measured by the BART had a significant association with alcohol dependence severity, while it had no significant association with ELS. Substantial evidence support the relationship between risk taking propensity and alcohol problems^26,27^, but the findings regarding its relationship with childhood trauma have been inconsistent^28,29^. A impulsivity study in young adults showed that self-reported scales of impulsivity and laboratory-based measures of risk taking such as the BART are differentially associated with ELS, in which subjects with childhood abuse showed significantly less risk-taking (fewer adjusted mean number of pumps) on the BART compared to those without experience of abuse^29^. As the authors mentioned, the finding on tasks such as BART may be influenced by hypervigilance or individuals’ state-dependent characteristics during the experiment rather than the actual impulsivity trait. Another possibility is that risk-taking propensity may be more affected by genetic factors rather than environmental factors such as childhood adverse experiences. A longitudinal twin genetic study of risk taking measured by the BART reported 55% heritability in males at age 14^30^. Further research is needed to confirm the relationship between risk taking and childhood trauma.

In the model of social onset, only the reflection impulsivity dimension, defined as the predisposition to act quickly without adequate evidence before decision-making, was a significant partial mediator in the relationship between childhood trauma and onset of social problems due to alcohol consumption. The reflection impulsivity dimension also showed the strongest association with alcohol dependence severity among impulsivity dimensions. Although the specific role of reflection impulsivity is not well known in alcohol use, a few studies in young binge drinker showed its association with binge drinking^31,32^. Our results suggest that exposure to ELS may confer vulnerability toward reflection impulsivity, through which they tend to choose alcohol drinking without full contemplation of harmful consequences as a means of self-regulation and avoidance to stressors. This is supported by brain neurobiology in which ELS exacerbates impulsivity and self-dysregulation particularly during adolescence, a critical developmental period of prefrontal circuits and executive functions, possibly leading to early initiation of substance^25,33,34^. Since early initiation of alcohol drinking is related to subsequent risky behaviors^35^ and increased risk and chronic relapse of later-life alcohol dependence^36^, special efforts targeting impaired decision-making for individuals exposed to severe childhood trauma would be important.

There are some limitations of this study that should be noted. First, the present study with cross-sectional design in the patient sample with alcohol dependence cannot draw accurate conclusions regarding the causal relationship between childhood trauma, impulsivity and alcohol problems. For example, the relationship between impulsivity and alcohol use may be bidirectional, or they may have a shared genetic liability. Future longitudinal studies in individuals with childhood trauma are required to better establish causality and directions in their relationships. Second, data of “social onset” of hazardous drinking were collected only through self-questionnaire, which raises concerns about potential biases including recall accuracy and social desirability bias. Corroborating self-reported data with collateral information obtained from relatives and medical records would be helpful to enhance data validity in future studies. Third, we did not consider potential confounders such as recent stressors which could have influenced impulsivity level. Fourth, the study population included only male patients with alcohol dependence, which limits the generalizability of the findings to females. Considering gender differences in clinical characteristics of alcohol use disorder as well as impulsivity, future studies in a larger sample with male and female groups are needed to determine whether the relationship between early life stress, impulsivity and alcohol dependence severity can be observed across gender or whether there is a moderating effect of gender in the path.

In conclusion, this study showed that specific impulsivity dimensions have partial mediating effects in the associations between ELS and severity and onset of harmful alcohol drinking in patients with alcohol dependence. Among impulsivity dimensions, reflection impulsivity was the most significant factor affecting symptom severity and social onset of alcohol dependence. Our findings imply that stabilizing vulnerabilities such as reflection impulsivity via intervention programs that target impulsivity in young individuals with childhood trauma may be helpful in delaying the onset of harmful alcohol drinking and prevent alcohol use disorder. Future longitudinal studies in larger sample with childhood trauma exposures are needed to establish causal relationships and the underpinning mechanism of multi-dimensional impulsivity in the clinical course of alcohol use disorder.

## Methods

### Participants and procedure

A total of 330 male Korean patients with alcohol dependence were recruited from 16 mental hospitals with alcohol dependence clinics. All participants were patients admitted to psychiatric in-patient wards for management of alcohol withdrawal syndrome and rehabilitation, who had been abstinent from alcohol for at least 7 days prior to participation in the study. Abstinence was defined as abstinent from alcohol by surveillance of the medical staff when there was no sign of acute intoxication and withdrawal symptoms of alcohol observed by a psychiatrist for at least seven days. All patients were diagnosed with alcohol dependence by trained psychiatrists according to the Diagnostic and Statistical Manual of Mental Disorders (DSM)-IV criteria^37^. All patients were asked to answer standardized questions on socio-demographic characteristics, including age, years of education, marital status, occupation, average monthly income and height/weight and information regarding alcohol consumption. For “social onset” of hazardous alcohol drinking, they were asked to report the earliest age when social impairments in the patient’s life, such as interpersonal, occupational, or legal problems, developed due to alcohol consumption through self-questionnaire.

The exclusion criteria were as follows: (1) presence of physical or mental illnesses which could interfere with task performance; (2) history of other substance dependence in the last six months; (3) a score of less than 26 on the Korean version of Mini Mental State examination. All participants provided written informed consent prior to the beginning of this study. The study protocol was approved by the Institutional Review Board of Severance Hospital and all methods of this study were carried out in accordance with the approved guidelines.

### Assessment of childhood sexual abuse, maltreatment and parental conflicts

To evaluate childhood sexual abuse, the “sexual abuse” section of the Childhood Maltreatment Scale was used^38^. The scale is composed of 8 items which measure experience of minor sexual violence such as physical touch and verbal sexual abuse and 2 items which measure experience of severe sexual violence such as oral sex and sexual intercourse. On a six-point Likert scale, each item measures the frequency of such sexual abuse before the age of 18 (0 = never, 1 = it happened once, 2 = 2 times, 3 = 3–5 times, 4 = 6–10 times, 5 = more than 11 times).

Besides sexual abuse, to evaluate other forms of childhood maltreatment or adverse events, the modified, Korean version of the Parent-Child Conflict Tactics Scale (mPCCTS) was used, which is based on the Parent-Child Conflict Tactics Scale developed by Straus *et al*.^39,40^. The mPCCTS consists of 24 items; five items measure psychological maltreatment, nine measure physical maltreatment and ten measure neglect of children. On a six-point Likert scale, each item measures the frequency of such maltreatment during conflict with a parent, before the age of 12 (0 = never, 1 = it happened once, 2 = 2 times, 4 = 3–5 times, 8 = 6–10 times, 15 = 11–20 times and 25 = more than 25 times).

In addition, to evaluate the experience of parental conflict during childhood, the modified version of The Conflict Tactics Scale (mCTS) was used^41,42^. The scale was comprised of 10 items which measure verbal violence (1 item), minor physical violence (4 items) and severe physical violence (5 items). On a five-point Likert scale, each item measures the average frequency of such parental conflict before the age of 12 (0 = never, 1 = once or twice a year, 2 = once or twice a month, 3 = more than once a week, 4 = almost every day).

### Measures of harmful and hazardous alcohol drinking and alcohol dependence

The Alcohol Use Disorders Identification Test (AUDIT), a widely used 10-item scale of alcohol dependence, was used to assess the severity of problematic alcohol consumption^43,44^. Higher scores on the AUDIT reflect more problematic alcohol drinking. To measure alcohol-related craving, the Obsessive Compulsive Drinking Scale (OCDS) was used^45^. It is composed of 14 questions that represent two domains: the obsessive subscale for thoughts about drinking and the compulsive subscale for drinking behavior^46^. In addition, to assess the severity of alcohol dependence, the Alcohol Dependence Scale – Korean (ADSK) was applied. The ADS is a 25-item scale concerning alcohol use in the previous 12 months that measures alcohol withdrawal symptoms, impaired control over drinking, awareness of a compulsion to drink, increased tolerance to alcohol and salience of drink-seeking behavior^47^.

### Assessment of multi-dimensional impulsivity

#### UPPS Impulsive Behavior Scale

The UPPS-P is a 59-item scale which represents 5 different dimensions of impulsivity: negative urgency, positive urgency, (lack of) premeditation, (lack of) perseverance and sensation seeking^19,48,49^. The items are scored on a scale ranging from 1 (disagree strongly) to 4 (agree strongly).

#### Barratt Impulsiveness Scale–Version 11 (BIS-11)

The BIS is a 30-item self-report assesses impulsivity through three sub-traits of attention impulsivity, motor impulsivity and non-planning impulsivity^50^. The items are scored on a scale ranging from 1 (rarely/never) to 4 (almost always/always). The BIS is one of the most commonly used self-report measure of impulsiveness.

#### Aggression

The Buss-Perry Aggression Questionnaire (BPAQ) is a self-report measure of aggression with four factors, which are physical aggression, verbal aggression, anger and hostility^51,52^. The total score on the BPAQ is indicative of the overall measures of anger and aggression.

#### Balloon Analogue Risk Task (BART)

The BART is a computerized behavioral measure of risk taking, during which the participants are rewarded for risky behavior up until further riskiness results in loss of the earned reward^53^. During the task, the participants could either inflate a balloon on a computer screen or end trial and move on to the next one. For each pump, the balloon inflated and the participants were rewarded with certain amount of money, which was saved in a temporary bank. The participants were informed that at a certain, random pump, the balloon would explode and the money in the temporary bank would be lost. Participants chose whether to inflate the balloon or to collect the money from the temporary bank to their permanent account, any time they wished before the balloon exploded. When the participant pops the balloon or collects the money, a new balloon would appear, for a total of 30 balloons. The participants did not collect real money but they were told to act as if it was real. Risk taking was measured by calculating the mean number of pumps in trials during which the balloons did not explode (adjusted mean pumps, AMP).

### Statistical analysis

Statistical analysis was conducted using Statistical Package for the Social Sciences version 24.0 and AMOS version 23.0 (SPSS Inc., Chicago, IL, USA).

To organize the various subscales of impulsivity measures into constructs that represent different facets of impulsivity, factor analysis was done with principal component method of factor extraction and with Varimax rotation for the following 13 variables: 5 subscales of UPPS, 3 subscales of BIS, 4 subscales of BPAQ and adjusted mean numbers of pumps on the BART. Factor scores were calculated for each factor using regression method. In addition, Pearson’s correlation was done with the factor scores of the reduced components and the variables regarding childhood maltreatment and alcohol use severity.

To estimated and test mediation effects, structural equation modeling was done with maximum likelihood using AMOS. The model included the latent variables that represent ELS, alcohol dependence severity or social onset and multi- dimensional impulsivity extracted from the factor analysis, which would mediate the former two latent variables. The latent variable “ELS” consisted of the variables mPCCTS, sexual abuse and mCTS. The latent variable “Alcohol Dependence Severity” consisted of the variables AUDIT, OCDS and ADSK. The latent variables representing the mediators regarding multi-dimensional impulsivity were created with factors that were reduced to the same impulsivity component during the factor analysis. All effect estimates are presented as unstandardized regression coefficients, as recommended by Preacher and Hayes^54^.

Statistical fit of the model was assessed using Comparative Fit Index (CFI) and Root Mean Square Error of Approximation (RMSEA) and the CFI values above 0.9 and RMSEA values less than 0.1 were considered as the indicator of good fit.

## Electronic supplementary material

Supplementary Information
