# Supplementary material for: Multidimensional impulsivity as a mediator of early life stress and alcohol dependence
Source: Sci Rep. 2018 Mar 7;8:4104. doi: 10.1038/s41598-018-22474-8 (PMC5841284; doi:10.1038/s41598-018-22474-8)
Supplement: Supplementary file 1 — Supplementary Information [file 41598_2018_22474_MOESM1_ESM.pdf]

Supplementary Information

Multidimensional impulsivity as a mediator of early life stress and alcohol dependence

Shin Tae Kim, BA [a], Syung Shick Hwang, MD [b], Hae Won Kim, MD PhD [a], Eun Hee Hwang, MS [a], Jaeil Cho, MD [c], Jee In Kang, MD PhD [a, d] †, Se Joo Kim, MD PhD [a, d] †

[a] Institute of Behavioral Science in Medicine, Yonsei University College of Medicine, Seoul, South Korea

[b] Graduate School, Yonsei University College of Medicine, Seoul, South Korea

[c] Yonsei Soul Mental Health Clinic, Seoul, South Korea

[d] Department of Psychiatry, Yonsei University College of Medicine, Seoul, South Korea

† Corresponding author:

Se Joo Kim, M.D., Ph,D., & Jee In Kang, M.D., Ph.D.

Department of Psychiatry and Institute of Behavioral Science in Medicine, Yonsei University College of Medicine

50-1 Yonsei-ro, Seodaemun-gu, Seoul 03722, South Korea

Tel：+82-2-2228-1620, Fax：+82-2-313-0890

E-mail : kimsejoo@yuhs.ac (S.J. Kim), jeeinkang@yuhs.ac (J.I. Kang)

Supplementary Table S1. Correlations between observed variables and factor scores

	1	1.1	1.2	1.3	1.4	1.5	2	2.1	2.2	2.3	3	3.1	4	4.1	4.2	4.3	4.4	5	5.1	5.2	5.3	6	6.1	6.2	6.3	7
1. Refl. Impulsivity	-																									
1.1. UPPS: Per	.84**																									
1.2. UPPS: Pre	.77**	.66**																								
1.3. BIS: A	.82**	.55**	.51**																							
1.4. BIS: M	.76**	.56**	.47**	.62**																						
1.5. BIS: NP	.86**	.64**	.56**	.74**	.66**																					
2. Resp. Impulsivity	0	-0.1	-.22**	.12*	.29**	.21**																				
2.1. UPPS: NU	.41**	.31**	.20**	.34**	.51**	.48**	.71**																			
2.2. UPPS: PU	0.1	0.04	-0.06	.16**	.34**	.25**	.89**	.70**																		
2.3. UPPS: Sen	-.18**	-.18**	-.12*	-0.05	0.11	0.01	.72**	.33**	.51**																	
3. Risk Taking	0	-0.02	0.07	0.01	-0.01	-0.01	0	0.01	-0.04	0.04																
3.1 BART	0.03	0.02	0.08	0.02	0.03	0.01	0.01	0.03	0	0	.98**															
4. Aggression	0	0.07	.14*	-0.06	.22**	0.08	0	.27**	.15**	.21**	0	0.01														
4.1. BPAQ: PA	.19**	.15**	.16**	.14*	.30**	.25**	.18**	.42**	.30**	.29**	.12*	0.09	.79**													
4.2. BPAQ: VA	-.12*	-0.07	-0.01	-0.08	.13*	0.02	0.04	.20**	.14**	.24**	0.11	0.07	.80**	.55**												
4.3. BPAQ: A	.16**	.16**	.20**	0.09	.35**	.19**	.21**	.42**	.37**	.26**	-0.1	-0.04	.83**	.65**	.53**											
4.4. BPAQ: H	.18**	.19**	.18**	.12*	.34**	.24**	.24**	.41**	.34**	.21**	-.12*	-0.01	.75**	.53**	.46**	.66**										
5. Early life stress	.14*	0.08	.12*	.13*	.24**	.20**	.22**	.23**	.21**	.20**	-0.02	-0.02	.20**	.25**	0.11	.24**	.19**									
5.1. mPCCTS	.15*	0.09	.13*	.14*	.20**	.19**	.18**	.23**	.16**	.15**	0.01	0	.20**	.24**	.11*	.21**	.19**	.86**								
5.2. Sexual Abuse	0.02	-0.04	0.03	0.04	.11*	0.06	.12*	0.07	.12*	.15**	0.03	0.03	0.09	.13*	0.07	.12*	0.04	.74**	.48**							
5.3. mCTS	.17**	.13*	.12*	.14*	.25**	.23**	.22**	.24**	.21**	.19**	-0.09	-0.07	.18**	.22**	0.06	.23**	.21**	.77**	.52**	.30**						
6. Alcohol dependence severity	.50**	.38**	.31**	.43**	.48**	.53**	.26**	.50**	.27**	.16**	0.08	0.11	.26**	.36**	.20**	.32**	.33**	.32**	.31**	.17**	.27**					
6.1. AUDIT	.43**	.32**	.27**	.37**	.40**	.44**	.22**	.45**	.24**	.14*	.15*	.16**	.24**	.32**	.22**	.28**	.29**	.29**	.27**	.20**	.20**	.90**				
6.2. OCDS	.50**	.39**	.33**	.40**	.47**	.52**	.21**	.45**	.25**	.12*	0.03	0.05	.23**	.31**	.15**	.28**	.27**	.27**	.24**	.12*	.27**	.89**	.70**			
6.3. ADS	.44**	.32**	.25**	.38**	.42**	.46**	.26**	.44**	.25**	.17**	0.04	0.07	.25**	.33**	.17**	.29**	.33**	.30**	.31**	.14**	.25**	.90**	.74**	.70**		
7. Social Onset	-0.11	-.15**	-.14*	-0.06	-.11*	-.22**	-0.11	-.17**	-0.1	-0.08	-0.03	-0.04	-0.09	-.13*	-0.01	-0.09	-.12*	-.21**	-.17**	-.12*	-.21**	-.24**	-.25**	-.21**	-.19**	-

Refl. Impulsivity: Reflection Impulsivity; Resp. Impulsivity: Response Impulsivity-Sensation Seeking; Child-Trauma: Childhood Trauma; UPPS: UPPS Impulsive Behavior Scale; BIS: Barratt Impulsiveness Scale; BPAQ: Buss-Perry Aggression Questionnaire; Pre: Lack of Pre-planning; Per: Lack of Perseverance; A: Attentional; M: Motor; NP: Non-planning; NU: Negative Urgency; PU: Positive Urgency; Sen: Sensation Seeking; BART: Balloon Analogue Risk Task; PA: Physical Aggression; VA: Verbal Aggression; A: Anger; H: Hostility; PA: Parental; AUDIT: Alcohol Use Disorders Identification Test; OCDS: Obsessive Compulsive Drinking Scale; ADS: Alcohol Dependence Scale

*p<.05

**p<.01

Supplementary Table S2a. Standardized regression weights for the mediation model A

Parameter	β	P
Early Life Stress→Response Impulsivity	0.294	<0.01
Early Life Stress→Reflection Impulsivity	0.24	<0.01
Early Life Stress→Aggression	0.31	<0.01
Early Life Stress→ Alcohol Dependence Severity	0.188	<0.05
Response Impulsivity→ Alcohol Dependence Severity	0.194	<0.05
Reflection Impulsivity→ Alcohol Dependence Severity	0.416	<0.01
Aggression→ Alcohol Dependence Severity	0.148	<0.05
BART→ Alcohol Dependence Severity	0.099	<0.05
Early Life Stress→Childhood Maltreatment	0.861	<0.01
Early Life Stress→Parental Conflict	0.611	<0.01
Early Life Stress→Sexual Abuse	0.538	<0.01
Response Impulsivity→UPPS: NU	0.938	<0.01
Response Impulsivity→UPPS: PU	0.743	<0.01
Response Impulsivity: UPPS: Sen	0.392	<0.01
Reflection Impulsivity: UPPS: Per	0.732	<0.01
Reflection Impulsivity→BIS: A	0.803	<0.01
Reflection Impulsivity→UPPS: Pre	0.654	<0.01
Reflection Impulsivity→BIS: M	0.762	<0.01
Reflection Impulsivity→BIS: NP	0.895	<0.01
Aggression→BPAQ: A	0.856	<0.01
Aggression→BPAQ: VA	0.629	<0.01
Aggression→BPAQ: PA	0.766	<0.01
Aggression→BPAQ: H	0.751	<0.01
Alcohol Dependence Severity→AUDIT	0.853	<0.01
Alcohol Dependence Severity→OCDS	0.822	<0.01
Alcohol Dependence Severity→ADS	0.855	<0.01

UPPS: UPPS Impulsive Behavior Scale; BIS: Barratt Impulsiveness Scale; BPAQ: Buss-Perry Aggression Questionnaire; Pre: Lack of Pre-planning; Per: Lack of Perseverance; A: Attentional; M: Motor; NP: Non-planning; NU: Negative Urgency; PU: Positive Urgency; Sen: Sensation Seeking; BART: Balloon Analogue Risk Task; PA: Physical Aggression; VA: Verbal Aggression; A: Anger; H: Hostility; AUDIT: Alcohol Use Disorders Identification Test; OCDS: Obsessive Compulsive Drinking Scale; ADS: Alcohol Dependence Scale

Supplementary Table S2b. Standardized regression weights for the mediation model B

Parameter	β	P
Early Life Stress→Social Onset	-0.186	<0.05
Early Life Stress→Reflection Impulsivity	0.239	<0.01
Reflection Impulsivity→Social Onset	-0.143	<0.05
Early Life Stress→Childhood Maltreatment	0.856	<0.01
Early Life Stress→Parental Conflict	0.614	<0.01
Early Life Stress→Sexual Abuse	0.541	<0.01
Reflection Impulsivity→UPPS: Per	0.743	<0.01
Reflection Impulsivity→BIS: A	0.805	<0.01
Reflection Impulsivity→UPPS: Pre	0.673	<0.01
Reflection Impulsivity→BIS: M	0.751	<0.01
Reflection Impulsivity→BIS: NP	0.887	<0.01

UPPS: UPPS Impulsive Behavior Scale; BIS: Barratt Impulsiveness Scale; Pre: Lack of Pre-planning; Per: Lack of Perseverance; A: Attentional; M: Motor; NP: Non-planning; AUDIT: Alcohol Use Disorders Identification Test; OCDS: Obsessive Compulsive Drinking Scale; ADS: Alcohol Dependence Scale
